# Association Between DNA Damage Response, Fibrosis and Type I Interferon Signature in Systemic Sclerosis

**DOI:** 10.3389/fimmu.2020.582401

**Published:** 2020-10-02

**Authors:** Nikolaos I. Vlachogiannis, Maria Pappa, Panagiotis A. Ntouros, Adrianos Nezos, Clio P. Mavragani, Vassilis L. Souliotis, Petros P. Sfikakis

**Affiliations:** ^1^First Department of Propaedeutic Internal Medicine, National and Kapodistrian University of Athens Medical School, Athens, Greece; ^2^Joint Academic Rheumatology Program, National and Kapodistrian University of Athens Medical School, Athens, Greece; ^3^Department of Physiology, School of Medicine, National and Kapodistrian University of Athens, Athens, Greece; ^4^Institute of Chemical Biology, National Hellenic Research Foundation, Athens, Greece

**Keywords:** systemic sclerosis (scleroderma), type I interferon (IFN), DNA damage response and repair network, oxidative stress, fibrosis

## Abstract

Increased endogenous DNA damage and type I interferon pathway activation have been implicated in systemic sclerosis (SSc) pathogenesis. Because experimental evidence suggests an interplay between DNA damage response/repair (DDR/R) and immune response, we hypothesized that deregulated DDR/R is associated with a type I interferon signature and/or fibrosis extent in SSc. DNA damage levels, oxidative stress, induction of abasic sites and the efficiency of DNA double-strand break repair (DSB/R) and nucleotide excision repair (NER) were assessed in peripheral blood mononuclear cells (PBMCs) derived from 37 SSc patients and 55 healthy controls; expression of DDR/R-associated genes and type I interferon–induced genes was also quantified. Endogenous DNA damage was significantly higher in untreated diffuse or limited SSc (Olive tail moment; 14.7 ± 7.0 and 9.5 ± 4.1, respectively) as well as in patients under cytotoxic treatment (15.0 ± 5.4) but not in very early onset SSc (5.6 ± 1.2) compared with controls (4.9 ± 2.6). Moreover, patients with pulmonary fibrosis had significantly higher DNA damage levels than those without (12.6 ± 5.8 vs. 8.8 ± 4.8, respectively). SSc patients displayed increased oxidative stress and abasic sites, defective DSB/R but not NER capacity, downregulation of genes involved in DSB/R (MRE11A, PRKDC) and base excision repair (PARP1, XRCC1), and upregulation of apoptosis-related genes (BAX, BBC3). Individual levels of DNA damage in SSc PBMCs correlated significantly with the corresponding mRNA expression of type I interferon–induced genes (IFIT1, IFI44 and MX1, *r*=0.419-0.490) as well as with corresponding skin involvement extent by modified Rodnan skin score (*r*=0.481).** **In conclusion, defective DDR/R may exert a fuel-on-fire effect on type I interferon pathway activation and contribute to tissue fibrosis in SSc.

## Introduction

The pathogenesis of systemic sclerosis (SSc) is a complex interplay of immune system activation, tissue fibrosis, and vasculopathy (1–4). The chronic proinflammatory environment of SSc has been associated with genomic instability (5) as evidenced by high rates of lymphocyte chromosomal abnormalities and the presence of cytogenetic micronuclei. As a result, accumulation of endogenous DNA damage, irrespective of disease subtype or drug treatment, occurs (6). Major sources of endogenous DNA damage include, among others, oxidative stress mediated mainly by reactive oxygen species (ROS).

Oxidative stress can induce single-strand DNA breaks (SSBs) that can be converted to cytotoxic double-strand DNA breaks (DSBs) when replication forks come across SSBs. SSc patients are indeed characterized by increased ROS production in the skin, visceral fibroblasts, and endothelial cells (7, 8). In turn, ROS can activate endothelial cells, leading to vascular hyper-reactivity, endothelial cell apoptosis, and impaired angiogenesis (7). Moreover, ROS act on differentiation and proliferation of fibroblasts and promote synthesis of extracellular matrix proteins leading to fibrosis (9). At the same time, ROS promote an autoimmune response through the genesis of neoepitopes and the activation of B and T lymphocytes and macrophages (7).

To protect against these genotoxic insults, a well-organized mechanism, namely the DNA damage response and repair (DDR/R) network, has been evolved in mammalian cells. DDR/R is a hierarchically structured mechanism that determines the cell’s ability to repair DNA damage or to undergo apoptosis (10). It is triggered by the detection of DNA lesions through specific sensors, and the next step is the initiation of a signal transduction cascade, which activates DNA repair mechanisms, cell cycle control, and apoptosis (11). The DDR/R network and the immune response act synergistically to enhance cellular defenses, thus protecting multicellular integrity from both exogenous and endogenous threats. Accumulating evidence suggests that aberrant activation of each one of these networks often leads to chronic and potentially fatal systemic autoimmune disease (11).

On the other hand, an aberrant immune response predominated by type I interferon (IFN) has been constantly shown in SSc (12–20). The presence of a prominent type I IFN signature has been associated with SSc severity, including the presence of lung fibrosis (13, 18) and pulmonary arterial hypertension (16), and type I IFN pathway activation has also been detected in the skin of SSc patients (21). However, the origin of the chronically elevated type I IFN in SSc remains unknown. Of interest, recent experimental evidence suggests a strong interplay between the DDR/R network and activated immune response (22–27). For example, oxidized DNA, i.e., from UV-induced damage or from ROS released during cell death, is shown to activate the innate immune receptor stimulator of interferon genes (STING) although “normal” DNA does not, which is explained by resistance of oxidized DNA to TREX1 degradation, thus accumulating in the cytoplasm (23).

Based on the above, herein, we tested the hypothesis that deregulated DDR/R in patients with SSc may be associated with the type I IFN signature and contribute to tissue fibrosis, the hallmark of this devastating disease.

## Materials and Methods

### Patients

We recruited 32 consecutive patients with SSc who had not previously received cytotoxic treatment, 5 consecutive patients treated with cyclophosphamide [for severe interstitial lung disease as recommended in the EULAR recommendations for the treatment of SSc (28)] (**Supplementary Table 1**), and 55 apparently healthy controls (HCs) with no personal or family history of systemic autoimmune disease (age- and sex-matched; *P*=0.48 and *P*=0.15, respectively). Seven SSc patients were characterized as very early diagnosis of SSc (VEDOSS), having Raynaud’s phenomenon with SSc marker autoantibodies and/or typical nailfold capillaroscopic findings and no manifestations other than puffy fingers (29, 30). The study was approved by the “Laiko” Hospital Ethical Committee, and all participants provided informed consent. Exclusion criteria for both groups included cancer, severe heart or renal disease, and active or recent (last 2 weeks) infection.

### Cell Isolation

Peripheral blood mononuclear cells (PBMCs) were isolated using Ficoll gradient centrifugation as previously described (31). Cells were resuspended in freezing medium [90% fetal bovine serum (FBS), 10% dimethyl sulfoxide (DMSO)] or lysed in TRIzol (Thermo Fisher Scientific, USA) and stored at -80°C until further processing.

### DNA Damage Measurements

Levels of endogenous DNA damage in PBMCs were assessed by single-cell gel electrophoresis (comet assay) measuring SSBs and/or DSBs under alkaline conditions as previously described (27).

### Oxidative Stress Measurements and Detection of Abasic Sites

Basal oxidative stress was measured using a luminescence-based system that detects and quantifies total glutathione (GSH), oxidized glutathione (GSSG), and the GSH/GSSG ratio according to manufacturer’s experimental protocol (GSH/GSSG-Glo™ Assay, Promega). The endogenous levels of abasic sites were evaluated using the OxiSelect Oxidative DNA Damage Quantitation Kit (AP-sites) according to the manufacturer’s experimental protocol.

### Repair Capacity of DNA Double-Strand Breaks (DSB/R)

Freshly isolated PBMCs were treated with 100 μg/ml melphalan for 5 min at 37°C in complete RPMI-1640 medium supplemented with 10% FBS, 100 units/ml penicillin, 100 μg/ml streptomycin, and 2 mmol/l L-glutamine. Cells were subsequently incubated in drug-free medium for various times (0–24 h), adhered to a coverslip, fixed, and stored at -80°C until the analysis of γH2AX. Immunofluorescence antigen staining and confocal laser scanning microscopy for the analysis of γH2AX foci (H2AX phosphorylated at Ser139; #9718T, Cell Signaling Technology) was performed as previously described (32).

### Nucleotide Excision Repair Measurement

Freshly isolated PBMCs were resuspended in PBS and irradiated with UVC with a total dose of 5 J/m^2^. Cells were then centrifuged and passed in RPMI medium containing 10% FBS and 100 units/ml penicillin and 100 μg/ml streptomycin and were incubated in a humidified CO_2_-incubator for 1, 2, and 6 h. Cells were then collected in freezing medium (90% FBS, 10% DMSO) and stored at -80°C until further processing.

### RT^2^ Profiler™ PCR Array

RNA extraction using the Qiagen RNeasy Mini Kit (QIAGEN), reverse transcription by the RT^2^ First Strand kit (QIAGEN), PCR array analysis using the RT² Profiler™ PCR Array (QIAGEN) of 84 genes related to the DNA damage signaling pathway (**Supplementary Table 2**), and data analysis by the RT^2^ Profiler PCR Array Data Analysis Webportal (https://geneglobe.qiagen.com/gr/analyze/) were performed as previously described (32).

### RNA Extraction, Reverse Transcription, and Type I IFN Score Calculation

RNA was extracted with the use of TRIzol Reagent (Thermo Fisher Scientific, USA) according to the manufacturer’s instructions. The quantity and quality of RNA samples were spectrophotometrically tested (Biospec Nano, Japan). One microgram of total RNA was reverse transcribed into cDNA with Superscript III (Thermo Fisher Scientific, USA). Complementary DNA samples were diluted 1:10 with nuclease-free water (Qiagen, Germany) immediately after synthesis and stored at -20°C. Quantitative real-time polymerase chain reaction (qRT-PCR) was used to quantify specific cDNAs using the Bio-Rad IQ5 thermocycler and the KAPA SYBR FAST Supermix (KAPA Biosystems, South Africa). Briefly, genes preferentially induced by type I IFNs according to recent data (12, 33) were selected and included the following: myxovirus (influenza virus) resistance 1 (MX-1), IFN-induced protein with tetratricopeptide repeats 1 (IFIT-1) and IFN-induced protein 44 (IFI44). As an internal control and normalization gene, we used the glyceraldehyde phosphate dehydrogenase (GAPDH). Specific primers for the selected genes are displayed in **Supplementary Table 3**. The reaction was carried out in a total volume of 20 *μ*L per reaction. The reaction mixture included 2 *μ*L of template cDNA, 0.2 *μ*M of each primer, 10 μl 2× KAPA SYBR FAST SuperMix (KAPA Biosystems, South Africa), and sterile water. The amplification protocol started with 95°C for 4 min followed by 40 cycles at 95°C for 5 s and 63°C for 30 s. To assess product specificity, amplicons were checked by melting curve analysis. Melting curves were generated from 65°C to 95°C with increments of 0.5°C/cycle for 15 s at each cycle, and all inconsistent results were discarded. Then, the threshold values were recorded for each sample in the logarithmic portion of the amplification curve. All reactions were performed in duplicate. In addition, a reference sample was included in each PCR plate to ensure normalization across experiments. Type I IFN score was calculated as previously described (34, 35).

### Statistical Analysis

Distribution of variables was examined by D’Agostino-Pearson and Shapiro-Wilk tests. Continuous variables are presented as mean ± SD. Pairwise comparisons were performed with the use of independent samples *t*-test or Mann-Whiney U test when normal distribution did not apply, and differences in categorical variables were examined by chi-square test. The Kruskal-Wallis test was used for groupwise differences among more than 2 groups. Correlations were examined with the use of Pearson’s correlation coefficient or the nonparametric Spearman’s test. Statistical significance was set at *a*=0.05. Statistical analysis was performed in SPSS v.26 and Graphpad Prism 7.

## Results

### Increased Endogenous DNA Damage in SSc

**Figure 1:**
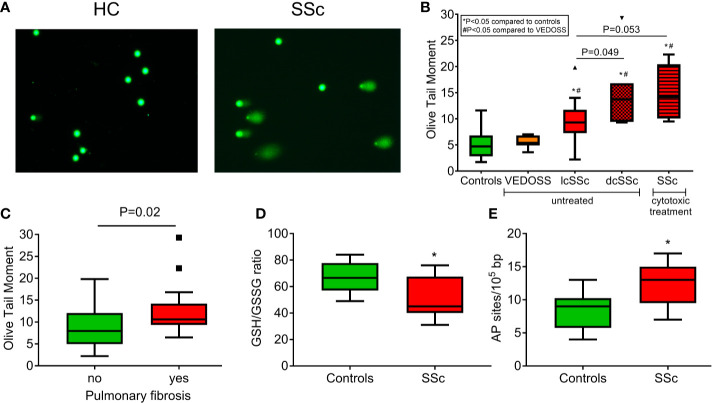
Increased endogenous DNA damage formation and accumulation in SSc. **(A)** Representative alkaline comet assay images of untreated peripheral blood mononuclear cells (PBMCs) from a control (HC) and an SSc patient. **(B)** Tukey boxplots representing the endogenous DNA damage levels (Olive tail moment arbitrary units) as assessed by alkaline comet assay in PBMCs from controls (*n*=55), VEDOSS (*n*=7), patients with limited (*n*=18) or diffuse (*n*=7) SSc currently on no cytotoxic therapy and patients with SSc under cytotoxic treatment (*n*=5). *P*-values are derived from Mann-Whitney U test; * P<0.05 compared to controls, ^#^P<0.05 compared to VEDOSS. **(C)** Tukey boxplots representing the endogenous DNA damage levels (Olive tail moment arbitrary units) as assessed by alkaline comet assay in PBMCs of SSc patients with (*n*=17) or without (*n*=20) pulmonary fibrosis. *P*-value is derived from Mann-Whitney U test. Tukey boxplots representing oxidative stress levels, expressed as the GSH/GSSG ratio **(D)** and the number of AP sites per 100,000 base pairs **(E)** in PBMCs of HC (*n*=10) and SSc patients (*n*=9). None of the patients were currently on cytotoxic therapy. *P<0.05 compared to controls.

First, the presence of endogenous DNA damage in PBMCs from patients with SSc was assessed using the alkaline comet assay measuring SSBs and/or DSBs (**Figure 1A**). Patients under cytotoxic treatment were examined separately, as genotoxic therapy has been shown to affect accumulation of DNA damage (27, 36). Endogenous DNA damage was significantly higher in untreated patients with diffuse (Olive tail moment; 14.7 ± 7.0) and limited SSc (9.5 ± 4.1) as well as in SSc patients treated with cytotoxic drugs (15.0 ± 5.4) (*P*<0.05 for all) but not in very early diagnosis of SSc (VEDOSS; 5.6 ± 1.2) compared to controls (4.9 ± 2.6) (**Figure 1B**), indicating that endogenous DNA damage accumulates in patients’ cells in the absence of known exogenous genotoxic insults. Moreover, patients with pulmonary fibrosis had higher DNA damage levels compared to those without pulmonary fibrosis (*P*=0.02; **Figure 1C**).

### Aberrant DDR/R Network in SSc Patients

Next, we examined whether DNA damage accumulation may be attributed to increased DNA damage formation and/or defective DNA repair—two conditions that are not mutually exclusive. We found that, compared with normal cells, PBMCs from SSc patients showed significantly higher levels of both oxidative stress (as indicated by the reduction of the GSH/GSSG ratio) and AP sites (both *P*<0.05; **Figures 1D, E**). These results suggest that the increased endogenous DNA damage measured in SSc cells may result, at least in part, from oxidative stress and/or the induction of abasic sites.

**Figure 2:**
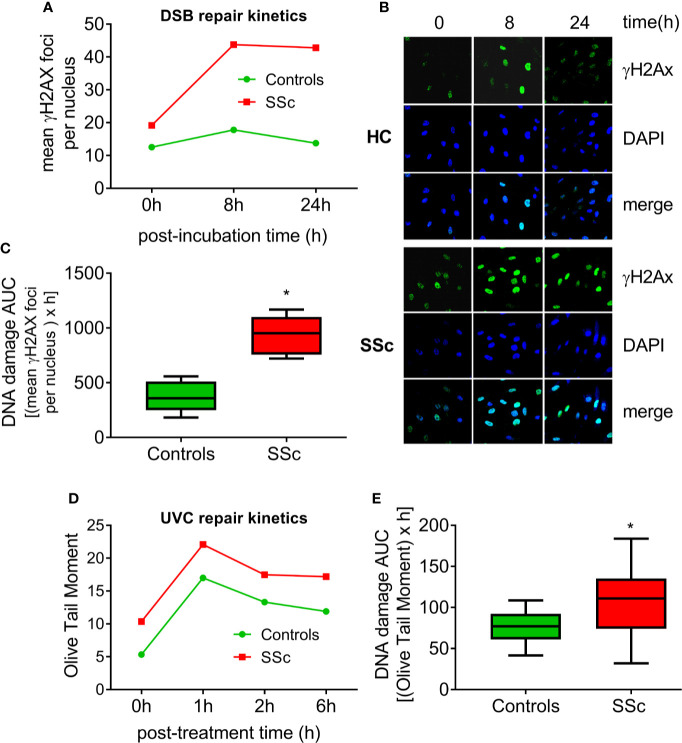
Deregulated DNA repair mechanisms in SSc. **(A)** Line-graphs showing the kinetics of γH2AX formation and removal 0–24 h after treatment of freshly isolated PBMCs from HC (*n*=9) and SSc patients (*n*=9) with melphalan. **(B)** Confocal microscopy images showing γH2AX staining at different time points after the ex vivo treatment of PBMCs from a representative HC and an SSc patient with melphalan. Upper images, immunofluorescence antigen staining; middle, cell nuclei labeled with DAPI; bottom, merged. **(C)** Tukey boxplots representing DNA DSB accumulation (γH2Ax immunofluorescence) 24 h after treatment with melphalan. **(D)** Line graphs showing levels of single- and double-strand DNA breaks (Olive tail moment) as assessed by alkaline comet assay in PBMCs of HC (*n*=24) and SSc patients (*n*=22) at baseline and 1, 2, and 6 h after ex vivo UVC irradiation of freshly isolated PBMCs with 5J/m^2^. **(E)** Tukey boxplots representing DNA SSB and DSB accumulation (alkaline comet assay) 6 h after treatment of PBMCs with 5J/m^2^ UVC. *P<0.05 compared to controls.

Moreover, we examined the efficacy of two main DNA repair mechanisms, namely DNA DSB/R and nucleotide excision repair (NER). To study the DSB/R, PBMCs were treated with the genotoxic drug melphalan as a tool to induce DSBs, and γH2AX foci levels were examined using immunofluorescence staining and confocal microscopy. Peak γH2AX foci levels were observed within 8 h of melphalan treatment and decreased thereafter (**Figures 2A, B**). SSc PBMCs showed significantly lower DSB/R capacity compared to control-derived PBMCs, resulting in higher DSB burden [expressed as the area under the curve for DNA adducts during the whole experiment (0–24 h)] in patients’ cells (*P*<0.001; **Figure 2C**).

In order to examine the capacity of NER, we irradiated freshly isolated PBMCs with UVC, which induces 6-4 photoproducts and cyclobutane pyrimidine dimers, both of which are exclusively repaired by NER. Peak DNA damage levels were observed 1 h after UVC irradiation. Thereafter, DNA damage levels were diminished with differences in the rates of adduct loss between SSc patients and HC being minor (**Figure 2D**). However, the DNA damage burden was significantly higher in SSc because of the higher levels of endogenous DNA damage observed in these patients (*P*=0.003; **Figure 2E**).

**Figure 3:**
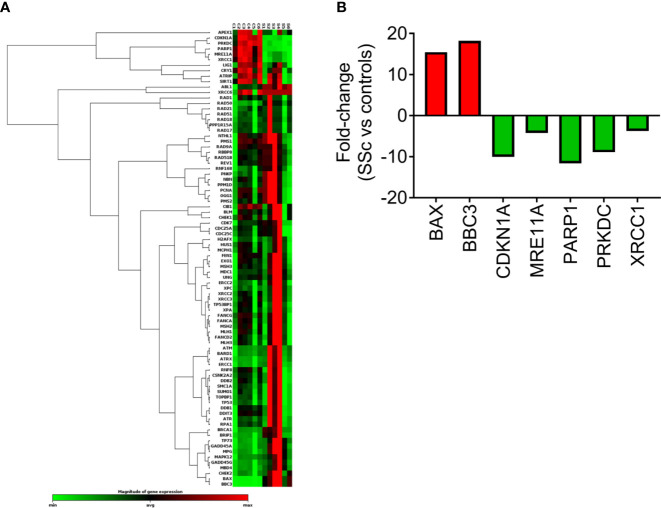
Transcriptional profiling of central DDR/R-associated genes. **(A)** Hierarchical clustergram of 84 DDR/R-associated genes in untreated PBMCs from 6 HCs and 6 SSc patients. **(B)** Genes demonstrating at least 2-fold difference in the transcript levels between SSc patients and HC. Gene acronyms are explained in **Supplementary Table 2**.

The expression of DDR/R-associated genes was also analyzed in PBMCs from 6 SSc patients at baseline and 6 controls by RT^2^ Profiler™ PCR Array (**Figure 3A**). Of 84 genes examined (**Figure 3A**, **Supplementary Table 2**), a total of 7 genes representing several non-mutually exclusive categories demonstrated at least a two-fold difference in gene expression between SSc patients and HC (**Figure 3B**). Particularly, the downregulated genes were categorized into DSB/R (MRE11A, PRKDC; 4- to 9-fold decrease in SSc vs. controls) and base excision repair (PARP1, XRCC1; 4- to 12-fold decrease in SSc vs. controls), and apoptosis-related genes were upregulated (BAX, BBC3; 15- to 18-fold increase in SSc vs. controls) (**Figure 3B**). The CDKN1A (p21) gene, which is implicated in the cell cycle arrest at the G1 checkpoint, was also downregulated in SSc patients (10-fold decrease) (**Figure 3B**).

### Association of Endogenous DNA Damage With Type I IFN in SSc PBMCs

**Figure 4:**
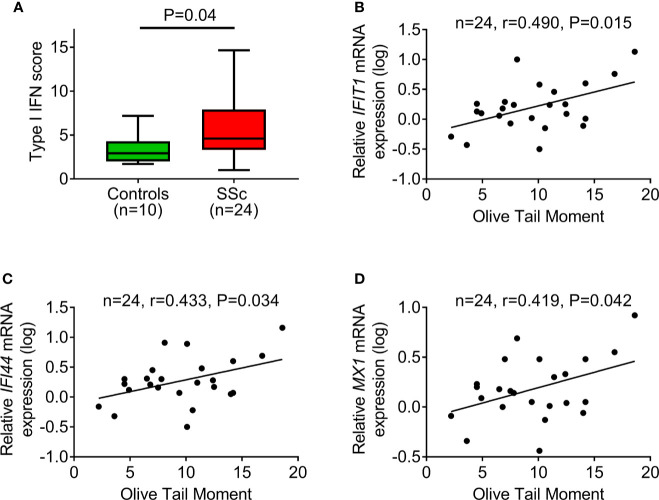
DNA damage accumulation in association with type I IFN pathway activation. **(A)** Tukey boxplots showing the type I IFN score in PBMCs of controls (*n*=10) and SSc patients (*n*=24). For the calculation of type I IFN score, the relative mRNA expression of 3 type I IFN–induced genes (IFIT1, IFI44, and MX1) was measured with RT-qPCR. *P*-value is derived from Mann-Whitney U test. Scatterplots showing the relationship between endogenous DNA damage levels (Olive tail moment) and mRNA expression of the type I IFN-induced genes IFIT1 **(B)**, IFI44 **(C)**, and MX1 **(D)** in PBMCs of SSc patients. Correlation coefficients are derived from Pearson’s test.

Next, the PBMC type I IFN score was calculated as a composite of three type I IFN–induced genes (IFIT1, IFI44, MX1) normalized to a housekeeping gene (GAPDH). In corroboration with previous reports (16), we detected a significantly increased type I IFN score in PBMCs of SSc patients compared to controls (*P*=0.04; **Figure 4A**). Interestingly, we observed that, in patients with SSc, endogenous DNA damage levels were associated with the corresponding expression levels of each one of the three type I IFN–induced genes analyzed (*r*=0.419–0.490, *P*<0.05 for all; **Figures 4B–D**), suggesting an interplay between the DDR/R network and the type I IFN response in SSc.

### Accumulation of Endogenous DNA Damage Is Proportional to Fibrosis Extent and Associates With Vascular Involvement

Finally, we examined the potential clinical implications of DNA damage accumulation in SSc. We observed that individual DNA damage levels in PBMCs strongly correlated with the extent of skin involvement in our patients assessed by the modified Rodnan skin score (mRSS) (*r*=0.481, *P*=0.003). Moreover, patients with pulmonary arterial hypertension (*n*=6) had significantly higher DNA damage levels compared to those without (*P*=0.004), and patients with digital ulcers (*n*=15) had a trend for higher endogenous DNA damage levels (*P*=0.099).

## Discussion

An aberrant immune response, including type I IFN pathway activation, is evident from the earliest phases of SSc, before the development of clinically overt fibrosis (12). The link between immune response and fibrosis has been intensively studied (2, 4); however, no treatment to date has managed to satisfactorily halt fibrosis progression in SSc patients. Thus, there is an imperative need to better understand the underlying pathogenetic mechanisms that may act as mediators or common denominators of immune activation and fibrosis in SSc in order to develop biomarkers for better patient risk stratification and to discover novel targets for targeted therapeutic approaches (37).

Herein, in line with previous data (6), we found increased endogenous DNA damage in PBMCs of SSc patients, regardless of disease subtype (diffuse or limited SSc) or drug treatment. These findings are not SSc-specific because our previous studies show that patients with other systemic autoimmune diseases, such as SLE and rheumatoid arthritis, are also characterized by several-fold higher endogenous DNA damage compared to HCs (27, 32). Lupus nephritis patients showed much higher intrinsic DNA damage than SLE patients with quiescent disease, suggesting that DNA damage levels may be associated with disease activity (32). Moreover, our recent study on rheumatoid arthritis provides evidence that, in parallel with the reduction of disease activity, antirheumatic treatment results in decreased accumulation of DNA damage in the same patients (27). Regarding SSc, activated T lymphocytes that are detectable in both circulation and the affected skin and lung (38) may undergo extraordinarily rapid cell division in the course of aberrant immune responses leading to unusual genomic stress (5). Indeed, a previous study shows that activated mouse and human T cells display a pronounced DDR/R network *in vitro* and *in vivo*, thus inducing significant amounts of DNA breakage (39).

To understand the origin of the increased endogenous DNA damage in SSc, we evaluated the induction of oxidative stress and AP sites, which are critical endogenous processes that lead to the intracellular formation of SSBs and DSBs (27). We find that SSc patients exhibit increased oxidative stress and AP sites, which is in line with previous studies showing that oxidative stress is implicated in the development and perpetuation of SSc (7, 40). Indeed, several oxidative stress biomarkers, such as malondialdehyde (a marker of lipid peroxidation), nitric oxide, and endogenous nitric oxide inhibitor asymmetric dimethylarginine are increased in the blood of SSc patients (41). In contrast, antioxidative biomarkers, such as superoxide dismutase and vitamin C, are lower in SSc patients’ blood (7). Oxidative-induced posttranslational protein modifications, such as advanced oxidation protein products, are also increased in the plasma of SSc patients compared to non-SSc controls (7). Moreover, SSc patients have higher urinary levels of 8-oxo-2′-deoxyguanosine (8-oxodG) and isoprostanes that are produced *in vivo* by free radical–catalyzed peroxidation of arachidonic acid compared to controls (40). Fibroblasts extracted from either fibrotic or nonfibrotic skin of patients with SSc also show higher ROS levels as compared to skin fibroblasts from HC, suggesting that oxidative stress may be an early event in the disease pathogenesis (9, 42). In addition to fibroblasts, high levels of ROS have also been measured ex vivo in different cell types from SSc patients, including monocytes, T lymphocytes, and erythrocytes, compared to healthy donor cells (7). This excessive oxidative stress of SSc patients could also explain, at least in part, the increased levels of AP sites and DSBs that were found in our patients because ROS produce such types of DNA damage.

Because accumulation of DNA damage can be also mediated by defective DNA repair mechanisms, the repair efficiency of DSBs that represent the most lethal form of DNA damage and the efficiency of NER were evaluated in PBMCs from SSc patients. Although normal NER capacity was observed in the SSc patients analyzed, defects in the DSB repair mechanism resulting in the accumulation of DSBs were found. Moreover, we found that critical DSB repair–associated genes, such as MRE11A and PRKDC (also known as DNA-PK) were underexpressed in SSc patients, thus explaining, in part, the reduced DSB/R capacity. Previous studies show that repair proteins implicated in the DSB/R mechanism are targets for autoantibodies, including Ku, the poly(ADP-ribose) polymerase (PARP), the Meiotic Recombination 11 Homolog (Mre11), the Werner Syndrome RecQ Like Helicase (WRN), and the Poly(ADP-Ribose) polymerase (PARP), which are found in patients with SSc (43). Also, we found that genes involved in base excision repair (PARP1 and XRCC1) were downregulated in SSc compared with HC. Previous studies demonstrate that PARP-1 is downregulated in SSc by increased DNA methylation in the PARP-1 promoter region (44), and XRCC1 polymorphisms are associated with increased endogenous DNA damage and the presence of antinuclear and anticentromere antibodies in SSc patients (6). Genes involved in apoptosis (BAX and BBC3) are overexpressed in SSc patients versus HC, in accordance with other studies showing accelerated apoptosis in SSc lymphocytes (45). Indeed, previous data show that CD8^+^ T cells from patients with SSc are characterized by enhanced expression of Bax and increased apoptosis rates (46). Also, endothelial cell apoptosis is a primary event in the pathogenesis of SSc, and anti-endothelial cell antibodies seem to be involved in this apoptosis induction (47).

Further, we found that type I IFN–induced gene expression is elevated in peripheral blood cells of SSc patients as previously described (12, 14, 20). Although the presence of a prominent type I IFN response in the blood of patients with systemic autoimmune diseases was described more than 40 years ago (48), the origin of the aberrant immune response remains unknown. Herein, we show that individual DNA damage levels in PBMCs are strongly associated with type I IFN expression in the same cells. Most studies to date concur that damaged DNA may induce an aberrant immune response through the cGAS/STING pathway, leading to IRF-3 phosphorylation and, consequently, type I IFN pathway activation (11). Other IFN regulatory factors, such as IRF-5, -7, or -8, as well as the downstream signal transducer STAT4, have also been implicated in SSc pathogenesis (20), further supporting the central role of type I IFN in SSc. Of interest, activation of the IRF factors by damaged DNA may directly upregulate IFN-stimulated genes (such as the ones studied herein) initiating an “antiviral”-like innate immune response without affecting type I IFN levels, as previously shown in Trex1-deficient mice (49). Another pathway linking DNA damage to aberrant immune response is inflammasome activation (especially AIM2) (50), which may also contribute to the observed inflammatory phenotype in SSc and other systemic autoimmune diseases. A handful of studies have previously shown the potential of DNA damage accumulation to induce the type I IFN pathway (11, 22–25). More specifically, treatment of healthy donor-derived primary monocytes with etoposide, a chemotherapeutic agent that blocks topoisomerase II activity and leads to the accumulation of DSBs, has been shown to upregulate type I IFN–induced gene expression and type III IFNs (25). Similarly, defective ribonucleotide removal and accumulation of base lesions and chronic low-grade DNA damage has been shown to “prime” the innate immune response (24). Fibroblasts from Aicardi–Goutières syndrome and SLE patients with mutations in the DNA repair enzyme RNaseH2 produce increased levels of IFNβ upon stimulation with poly(I:C) ex vivo, a phenomenon that is enhanced by UVC irradiation (24). More importantly, oxidized DNA was shown to colocalize with the type I IFN–induced MX1 in skin biopsies of patients with SLE (23). Conversely, loss of immune homeostasis and prolonged inflammation generated by different sources (i.e., chronic infection, autoimmune disease, aging) drives the induction of the DDR/R network through the production of DNA damage (51).

Finally, we find a strong correlation between endogenous DNA damage levels in PBMCs with mRSS, suggesting potential biomarker characteristics for fibrosis extent in SSc. In line with this, urine 8-oxodG levels have been previously associated with the presence of pulmonary fibrosis on computerized tomography scan (40). Previous experimental evidence supports that oxidative DNA damage induces an ATM-mediated transcriptional suppression of the Wnt inhibitor factor 1 in fibroblasts of SSc patients resulting in increased collagen production and fibrosis (52). Of interest, stimulation of normal fibroblasts with SSc patient immunoglobulins or oxidative DNA-damaging agents was able to recapitulate the phenotype *in vitro*, connecting the aberrant immune response with fibrosis in a DDR-mediated mechanism (52). Similarly, ATM-induced phosphorylation of the receptor for advanced glycation endproducts (RAGE) was recently shown to be integral for efficient DSB/R to prevent DNA DSB accumulation, induction of cellular senescence, and tissue fibrosis (53). Of interest, RAGE-knockout mice, ATM-deficient mice, and other mouse models with persistent DSB signaling have been shown to develop pulmonary fibrosis (54, 55), suggesting that DSB accumulation may be a trigger of profibrotic pathways. In the same direction, accumulation of mitochondrial DNA damage in alveolar epithelial cells or myofibroblasts has been shown to promote the development of pulmonary fibrosis (56, 57). More importantly, development of pulmonary fibrosis has been observed among patients with Ataxia-Telangiectasia (mutation in the central DSB/R molecule ATM) (58), and telomerase dysfunction has been causatively linked with familial idiopathic pulmonary fibrosis (59). Of interest, the profibrotic role of self-DNA may be partially reversed by inhibition of cGAS (60), providing another link between DNA damage, immune activation, and fibrosis.

Limitations of the current study include the following: 1) Whether increased accumulation of DNA damage in peripheral blood correlates with SSc disease activity was not thoroughly studied. 2) Due to the study’s cross-sectional design, our findings do not propose a prognostic significance of DNA damage levels for fibrosis extent in SSc but rather suggest a relationship between the two mechanisms. 3) Our findings offer correlative evidence linking DNA damage with type I IFN response and fibrosis; however, no mechanisms are explored in this study. Future preclinical studies are warranted to examine the contribution of DNA damage to the aberrant immune response and profibrotic pathway activation observed in SSc.

To conclude, increased endogenous DNA damage in PBMCs of SSc patients is associated with fibrosis and may be explained by both increased DNA damage formation and defective DNA repair, which are associated with relevant gene expression downregulation. In addition, endogenous DNA damage individual levels also correlate with type I IFN-induced gene expression, suggesting a fuel-on-fire effect that may further propagate innate immune response in a vicious auto-loop and contribute to tissue fibrosis in SSc.

## Data Availability Statement

The raw data supporting the conclusions of this article will be made available by the authors, without undue reservation.

## Ethics Statement

The studies involving human participants were reviewed and approved by “Laiko” Hospital Ethical Committee. The patients/participants provided their written informed consent to participate in this study.

## Author Contributions

Study conception: NV, CM, VS, PS. Patient recruitment: NV, MP, PN, PS. Experiments: NV, MP, PN, AN, CM, VS. Drafting of manuscript: NV, VS, PS. Critical review of manuscript: all authors. All authors contributed to the article and approved the submitted version.

## Funding

Supported by ELKE grants (PS) of the National Kapodistrian University of Athens and PhD scholarship to MP by State Scholarships Foundation, Greece (IKY; 2018-050-0502-13136).

## Conflict of Interest

The authors declare that the research was conducted in the absence of any commercial or financial relationships that could be construed as a potential conflict of interest.
