# Supplementary material for: Association Between DNA Damage Response, Fibrosis and Type I Interferon Signature in Systemic Sclerosis
Source: Front Immunol. 2020 Oct 2;11:582401. doi: 10.3389/fimmu.2020.582401 (PMC7566292; doi:10.3389/fimmu.2020.582401)
Supplement: Supplementary file 1 [file Table_1.pdf]

SUPPLEMENTARY DATA

for

Association between DNA damage response, fibrosis and type I interferon signature in Systemic Sclerosis

Nikolaos I. Vlachogiannis,MD[1,2,*], Maria Pappa,MD[1,2,*], Panagiotis A. Ntouros,MD[1,2], Adrianos Nezos,PhD[3], Clio P. Mavragani,MD[2,3], Vassilis L. Souliotis,PhD[1,4], Petros P. Sfikakis,MD[1,2]

1. First Department of Propaedeutic Internal Medicine, National and Kapodistrian University of Athens Medical School, Athens, Greece

2. Joint Academic Rheumatology Program, National and Kapodistrian University of Athens Medical School, Athens, Greece

3. Department of Physiology, School of Medicine, National and Kapodistrian University of Athens, Athens, Greece

4. Institute of Chemical Biology, National Hellenic Research Foundation, Athens, Greece

*equal contribution

Corresponding author: Petros P. Sfikakis, MD; First Department of Propaedeutic Internal Medicine, School of Medicine, National and Kapodistrian University of Athens, 17 Agiou Thoma Street, 11527 Athens, Greece. *E-mail address*: psfikakis@med.uoa.gr

Running title: DNA Damage, Type I Interferon and Fibrosis

Table of Contents

- **Supplementary Table 1.** Demographics and disease characteristics of the study cohort
- **Supplementary Table 2**. RT² Profiler™ PCR Array Human DNA Damage Signaling Pathway: Gene list
- **Supplementary Table 3:** Type I Interferon inducible genes primer sequences for gene expression analysis.

Supplementary Table 1. Demographics and disease characteristics of the study cohort

	**SSc (n=37)**
Female gender, n(%)	34 (91.9)
Age, years	53.5±13.5
Disease duration, years; mean±SD (range)	5.9±7.6 (0-34)
Phenomenon Raynaud's, n(%)	37 (100)
VEDOSS/ limited SSc / diffuse SSc, n	7/ 19 / 11
Puffy fingers, n(%)	29 (78.4)
Digital ulcers, n(%)	15 (40.5)
Telangiectasia, n(%)	18 (48.6)
Pulmonary fibrosis, n(%)	17 (45.9)
Arthritis, n(%)	17 (45.9)
PAH defined by cardiac ultrasound, n(%)	6 (16.2)
mRSS VEDOSS/ limited SSc / diffuse SSc	0/3.3±2.8/13.7±10.3
ESR, mm/1st hour	23±18
ANA positive, n(%)	35 (94.6)
ACA positive, n(%)	10 (27.8)
Scl-70 positive, n(%)	19 (51.4)
Current immunomodulatory therapy, n(%)	5 (13.5)
Current ERA therapy, n(%)	8 (21.6)

Continuous variables are presented as mean±SD and categorical variables are presented as absolute count (valid percentage).

Abbreviations: VEDOSS: very early diagnosis of systemic sclerosis, SSc: systemic sclerosis, PAH: pulmonary arterial hypertension, mRSS: modified Rodnan skin score, ESR: erythrocyte sedimentation rate, ANA: antinuclear antibodies, ACA: anti-centromere antibodies, ERA: endothelin receptor antagonist.

Supplementary Table 2. RT² Profiler™ PCR Array Human DNA Damage Signaling Pathway: Gene list

No	Symbol	GeneBank	Description
1	ABL1	NM_005157	C-abl oncogene 1, non-receptor tyrosine kinase
2	APEX1	NM_080649	APEX nuclease (multifunctional DNA repair enzyme) 1
3	ATM	NM_000051	Ataxia telangiectasia mutated
4	ATR	NM_001184	Ataxia telangiectasia and Rad3 related
5	ATRIP	NM_032166	ATR interacting protein
6	ATRX	NM_000489	Alpha thalassemia/mental retardation syndrome X-linked
7	BARD1	NM_000465	BRCA1 associated RING domain 1
8	BAX	NM_004324	BCL2-associated X protein
9	BBC3	NM_014417	BCL2 binding component 3
10	BLM	NM_000057	Bloom syndrome, RecQ helicase-like
11	BRCA1	NM_007294	Breast cancer 1, early onset
12	BRIP1	NM_032043	BRCA1 interacting protein C-terminal helicase 1
13	CDC25A	NM_001789	Cell division cycle 25 homolog A (S. pombe)
14	CDC25C	NM_001790	Cell division cycle 25 homolog C (S. pombe)
15	CDK7	NM_001799	Cyclin-dependent kinase 7
16	CDKN1A	NM_000389	Cyclin-dependent kinase inhibitor 1A (p21, Cip1)
17	CHEK1	NM_001274	CHK1 checkpoint homolog (S. pombe)
18	CHEK2	NM_007194	CHK2 checkpoint homolog (S. pombe)
19	CIB1	NM_006384	Calcium and integrin binding 1 (calmyrin)
20	CRY1	NM_004075	Cryptochrome 1 (photolyase-like)
21	CSNK2A2	NM_001896	Casein kinase 2, alpha prime polypeptide
22	DDB1	NM_001923	Damage-specific DNA binding protein 1, 127kDa
23	DDB2	NM_000107	Damage-specific DNA binding protein 2, 48kDa
24	DDIT3	NM_004083	DNA-damage-inducible transcript 3
25	ERCC1	NM_001983	Excision repair cross-complementing rodent repair deficiency, complementation group 1 (includes overlapping antisense sequence)
26	ERCC2	NM_000400	Excision repair cross-complementing rodent repair deficiency, complementation group 2
27	EXO1	NM_130398	Exonuclease 1
28	FANCA	NM_000135	Fanconi anemia, complementation group A
29	FANCD2	NM_033084	Fanconi anemia, complementation group D2
30	FANCG	NM_004629	Fanconi anemia, complementation group G
31	FEN1	NM_004111	Flap structure-specific endonuclease 1
32	GADD45A	NM_001924	Growth arrest and DNA-damage-inducible, alpha
33	GADD45G	NM_006705	Growth arrest and DNA-damage-inducible, gamma
34	H2AFX	NM_002105	H2A histone family, member X

35	HUS1	NM_004507	HUS1 checkpoint homolog (S. pombe)
36	LIG1	NM_000234	Ligase I, DNA, ATP-dependent
37	MAPK12	NM_002969	Mitogen-activated protein kinase 12
38	MBD4	NM_003925	Methyl-CpG binding domain protein 4
39	MCPH1	NM_024596	Microcephalin 1
40	MDC1	NM_014641	Mediator of DNA-damage checkpoint 1
41	MLH1	NM_000249	MutL homolog 1, colon cancer, nonpolyposis type 2 (E. coli)
42	MLH3	NM_014381	MutL homolog 3 (E. coli)
43	MPG	NM_002434	N-methylpurine-DNA glycosylase
44	MRE11A	NM_005590	MRE11 meiotic recombination 11 homolog A (S. cerevisiae)
45	MSH2	NM_000251	MutS homolog 2, colon cancer, nonpolyposis type 1 (E. coli)
46	MSH3	NM_002439	MutS homolog 3 (E. coli)
47	NBN	NM_002485	Nibrin
48	NTHL1	NM_002528	Nth endonuclease III-like 1 (E. coli)
49	OGG1	NM_002542	8-oxoguanine DNA glycosylase
50	PARP1	NM_001618	Poly (ADP-ribose) polymerase 1
51	PCNA	NM_182649	Proliferating cell nuclear antigen
52	PMS1	NM_000534	PMS1 postmeiotic segregation increased 1 (S. cerevisiae)
53	PMS2	NM_000535	PMS2 postmeiotic segregation increased 2 (S. cerevisiae)
54	PNKP	NM_007254	Polynucleotide kinase 3'-phosphatase
55	PPM1D	NM_003620	Protein phosphatase, Mg2+/Mn2+ dependent, 1D
56	PPP1R15A	NM_014330	Protein phosphatase 1, regulatory (inhibitor) subunit 15A
57	PRKDC	NM_006904	Protein kinase, DNA-activated, catalytic polypeptide
58	RAD1	NM_002853	RAD1 homolog (S. pombe)
59	RAD17	NM_002873	RAD17 homolog (S. pombe)
60	RAD18	NM_020165	RAD18 homolog (S. cerevisiae)
61	RAD21	NM_006265	RAD21 homolog (S. pombe)
62	RAD50	NM_005732	RAD50 homolog (S. cerevisiae)
63	RAD51	NM_002875	RAD51 homolog (S. cerevisiae)
64	RAD51B	NM_133509	RAD51 homolog B (S. cerevisiae)
65	RAD9A	NM_004584	RAD9 homolog A (S. pombe)
66	RBBP8	NM_002894	Retinoblastoma binding protein 8
67	REV1	NM_016316	REV1 homolog (S. cerevisiae)
68	RNF168	NM_152617	Ring finger protein 168
69	RNF8	NM_183078	Ring finger protein 8
70	RPA1	NM_002945	Replication protein A1, 70kDa
71	SIRT1	NM_012238	Sirtuin 1
72	SMC1A	NM_006306	Structural maintenance of chromosomes 1A
73	SUMO1	NM_003352	SMT3 suppressor of mif two 3 homolog 1 (S. cerevisiae)
74	TOPBP1	NM_007027	Topoisomerase (DNA) II binding protein 1

75	TP53	NM_000546	Tumor protein p53
76	TP53BP1	NM_005657	Tumor protein p53 binding protein 1
77	TP73	NM_005427	Tumor protein p73
78	UNG	NM_003362	Uracil-DNA glycosylase
79	XPA	NM_000380	Xeroderma pigmentosum, complementation group A
80	XPC	NM_004628	Xeroderma pigmentosum, complementation group C
81	XRCC1	NM_006297	X-ray repair complementing defective repair in Chinese hamster cells 1
82	XRCC2	NM_005431	X-ray repair complementing defective repair in Chinese hamster cells 2
83	XRCC3	NM_005432	X-ray repair complementing defective repair in Chinese hamster cells 3
84	XRCC6	NM_001469	X-ray repair complementing defective repair in Chinese hamster cells 6

Supplementary Table 3: Type I Interferon inducible genes primer sequences for gene expression analysis.

Full Name	Primer	Accession Number	Forward Sequence (5'-3')	Reverse Sequence (5'-3')
Homo sapiens glyceraldehyde-3-phosphate dehydrogenase (GAPDH), mRNA	GAPDH	NM_002046	CAACGGATTTGGTCGTATT	GATGGCAACAATATCCACTT
Homo sapiens interferon-induced protein with tetratricopeptide repeats 1 (IFIT1), mRNA	IFIT1	NM_001548	CTCCTTGGGTTCGTCTATAAATTG	AGTCAGCAGCCAGTCTCAG
Homo sapiens myxovirus (influenza virus) resistance 1, interferon-inducible protein p78 (mouse) (MX1), mRNA	MX-1	NM_002462	TACCAGGACTACGAGATTG	TGCCAGGAAGGTCTATTAG
Homo sapiens interferon-induced protein 44 (IFI44), mRNA	IFI-44	NM_006417	CTCGGTGGTTAGCAATTATTCCTC	AGCCCATAGCATTCGTCTCAG
